# *Pulchragaricus rhodophyllus* gen. et sp. nov. (*Callistosporiaceae*, *Agaricales*) from Yunnan, China, Based on Morphological and Molecular Data

**DOI:** 10.3390/life16060899

**Published:** 2026-05-27

**Authors:** Sipeng Jian, Xinjing Xu, Tianwei Yang, Feng Gao, Jing Liu, Yiwei Fang, Wenzhu Ai, Chunxia Zhang

**Affiliations:** Yunnan Institute of Tropical Crops, Jinghong 666100, China; a1464993369@gmail.com (S.J.); 15198689253@163.com (X.X.); yangtianweizj@126.com (T.Y.); gaofeng19860704@outlook.com (F.G.); ljxsbn@126.com (J.L.); fangyiwei11@163.com (Y.F.); zhuge68168@163.com (W.A.)

**Keywords:** *Callistosporiaceae*, *Pulchragaricus*, multigene phylogeny, new genus, southwestern China

## Abstract

*Callistosporiaceae* is a recently established family within the suborder *Tricholomatineae*, encompassing tricholomatoid, collybioid or pleurotoid morphological forms. While most species of this family exhibit a saprotrophic lifestyle and have been predominantly documented in Europe and Americas, records from Asia remain comparatively sparse. In this study, *Pulchragaricus rhodophyllus* gen. et sp. nov., discovered in a *Pinaceae* and *Fagaceae* mixed forest in southwestern China, is described based on a comprehensive approach integrating both multigene phylogenetic analyses and morphological methods. A concatenated dataset comprising nuclear ribosomal DNA (ITS, LSU) and protein-coding genes (*rpb2*, *tef1-α*) provides robust statistical support for the placement of *Pulchragaricus* within *Callistosporiaceae*. Morphologically, this new taxon is characterized by a tomentose and yellowish-brown pileus, pink to pinkish lamellae, a solid and basally tapering stipe, broadly ellipsoid to ellipsoid basidiospores, sparse and subclavate cheilocystidia, and plentiful clamp connections. This discovery not only broadens the known diversity and distribution of the poorly documented Chinese *Callistosporiaceae*, but also offers a potential clue for understanding the evolutionary origins of the ectomycorrhizal symbiosis within the family.

## 1. Introduction

The family *Callistosporiaceae* Vizzini et al. was recently segregated from the *Biannulariaceae* Jülich (≡*Catathelasmataceae* Wasser). It currently comprises six genera: *Anupama* K.N.A. Raj et al., *Callistosporium* Singer, *Guyanagarika* Sánchez-García et al., *Macrocybe* Pegler & Lodge, *Pseudolaccaria* Vizzini et al., and *Xerophorus* (Bon) Vizzini et al. [1]. Members of this family are typically characterized by tricholomatoid, collybioid, or pleurotoid basidiomata, adnate to sinuate lamellae, and ellipsoid basidiospores. Ecologically, they exhibit predominantly saprotrophic lifestyles, with the notable exception of *Guyanagarika*. Phylogenetic analyses place *Callistosporiaceae* close to *Tricholomataceae* s. str. within the suborder *Tricholomatineae* Aime et al. [1,2]. To date, approximately 40 species have been described in this family [1,3,4].

Among them, *Callistosporium* is both the type genus and the most species-rich, containing around 25 collybioid to pleurotoid species that are primarily lignicolous and distributed across Europe, the Americas, and North Africa [1,5,6]. In stark contrast to the relatively small basidiomata of *Callistosporium*, the genus *Macrocybe* produces some of the largest basidiomata within the *Basidiomycota*. Pantropically distributed and comprising eight species [7], *Macrocybe* includes species such as *M. sardoa* Vizzini et al., a specimen of which was reported from India with a pileus exceeding 50 cm in diameter [8]. The remaining genera are mostly species-poor or monotypic, displaying terricolous or lignicolous habits. Notably, *Guyanagarika* represents the only verified ectomycorrhizal lineage within the *Callistosporiaceae*, forming symbiotic associations with *Pakaraimaea* Maguire & P.S. Ashton and *Dicymbe* Spruce ex Benth. & Hook.f. [3] (summarized in Table 1).

Records of *Callistosporiaceae* in China remain sparse. Although *Macrocybe gigantea* (Massee) Pegler & Lodge is cultivated on a large scale in China [11], the taxonomic literature concerning this species remains scarce. Recently, Xu et al. [5] described a new species, *Callistosporium subpetaloideum* J. Z. Xu & Yu Li, from Guizhou Province, thereby expanding the family’s known distribution. In a broader systematic context, Mou and Bau [12] erected the family *Asproinocybaceae* T. Bau & G.F. Mou to accommodate the genera *Asproinocybe* R. Heim and *Tricholosporum* Guzmán, which form the sister clade to *Callistosporiaceae*. In this study, we collected three noteworthy specimens from the mixed forest with *Fagaceae* Dumort. and *Pinaceae* Spreng. ex F. Rudolphi (*Pinus kesiya* var. *langbianensis* (A. Chev.) Gaussen ex Bui) in Pu’er City, southwestern Yunnan, China. Morphologically, these specimens exhibit tricholomatoid basidiomata with pinkish lamellae and robust stipes, superficially resembling *Agaricus* L. or *Leucopaxillus* Boursier. However, combined morphological and multigene phylogenetic analyses revealed them to represent a novel genus and species sister to *Guyanagarika*. The discovery of this putative ectomycorrhizal taxon provides a potential phylogenetic link, expanding our understanding of the family’s diversity in China and offering a tentative clue for the evolutionary origins of symbioses within the *Callistosporiaceae*.

## 2. Materials and Methods

### 2.1. Sampling and Morphological Studies

Details of the voucher specimens and sequences used in the phylogenetic analyses are listed in Table S1. Three specimens were collected from Pu’er City, Yunnan Province, China, during separate collecting trips in July and September (for the location map, please see Figure S2). Macromorphological descriptions were based on field notes and digital images. An Olympus TG-5 camera (Olympus Corporation, Tokyo, Japan) was used to record the colors of basidiomata in the field under natural light conditions. Hex triplet color codes were then extracted from the photographs with reference to ColorHexa (https://www.colorhexa.com, accessed on 24 February 2026) and are used here as approximate, visually matched descriptors, not as standardized colorimetric data. Traditional color standards, such as the *Methuen Handbook of Colour*, have known limitations, including edition-related discrepancies in hue [13]. Digital hex triplet color codes could provide a quantifiable and reproducible alternative that can facilitate cross-study comparisons. The size of basidiomata was defined with the standard proposed by Bas [14]. All voucher specimens have been deposited in the Cryptogamic Herbarium of the Kunming Institute of Botany, Chinese Academy of Sciences (KUN-HKAS).

For micromorphological observations, dried specimens were rehydrated and mounted in 5% KOH, deionized water, 1% aqueous Congo red, or Melzer’s reagent, and subsequently examined using a ZEISS Axio Scope A1 microscope (Zeiss Technology (Suzhou) Co., Ltd., Suzhou, China) under oil immersion at ×1000 magnification. Basidiospore measurements were obtained from images captured by a ZEN AxioCam ERc5s camera with the ZEN 2 imaging software (Carl Zeiss Microscopy GmbH, Jena, Germany). Note that basidiospore dimensions exclude the hilar appendix. It is worth noting that the specimen KUN-HKAS 154740 consists of a single immature basidioma, which did not provide sufficient mature basidiospores for reliable measurements. Therefore, the statistical data for basidiospores were derived entirely from specimens: KUN-HKAS 154741, which contained one mature basidioma, and KUN-HKAS 154742, which contained two mature basidiomata.

The notation [*n*/*m*/*p*] indicates that *n* basidiospores were measured from *m* basidiomata across *p* specimens. Measurements are presented as (a) b–c (d), where the range b–c encompasses at least 90% of the measured values, and the extremes are given in parentheses (a and d). L_m_ and W_m_ denote the mean length and width of the basidiospores (±standard deviation), respectively. The length/width ratio of an individual basidiospore is represented by Q, and the mean Q of all basidiospores (±standard deviation) is denoted by Q_m_.

### 2.2. DNA Extraction, Amplification, and Sequencing

Total genomic DNA was extracted from dried basidiomata using the cetyltrimethylammonium bromide (CTAB) method as described by Doyle and Doyle [15]. For phylogenetic analyses, four nuclear loci—including two ribosomal RNA regions and two protein-coding genes—were amplified: the internal transcribed spacer region (ITS), the large subunit of nuclear ribosomal RNA (LSU), the second largest subunit of RNA polymerase II (*rpb2*), and the translation elongation factor 1-alpha (*tef1-α*). ITS and LSU were selected for their broad utility in fungal phylogenetics and the availability of universal primers [16], whereas *rpb2* and *tef1-α* were included to provide a higher proportion of informative sites and robust nucleotide variation for resolving closely related lineages [1,12,17].

PCR amplifications were performed using the primer pairs ITS5/ITS4 for ITS, LR0R/LR5 for LSU, bRPB2-6F/bRPB2-7.1R for *rpb2*, and EF1-983F/EF1-1953R for *tef1-α*. Detailed amplification protocols followed previously established methods [18,19,20]. Specifically, PCR amplification was carried out in a 25 μL reaction mixture containing 2.5 μL of amplification buffer (with MgCl_2_), 2.5 μL of dNTP mixture (200 μM), 0.5 μL of Taq DNA polymerase (2.5 U/μL), 1 μL each of forward and reverse primer (10 μM), 0.5–2 μL of DNA template (depending on concentration), and ddH_2_O to adjust the final volume to 25 μL. The PCR protocol was executed as follows: initial denaturation at 95 °C for 5 min, 40 cycles of 95 °C for 30 s, annealing at an optimized temperature (*T_a_*) for 2 min, and 72 °C for 1 min, followed by a final extension at 72 °C for 10 min. The optimal *T_a_* was 52 °C for ITS, LSU, and *rpb2*, and 56 °C for *tef1-α*. The resulting PCR products were purified using a Gel Extraction and PCR Purification Combo Kit (spin-column, BioTeke Corporation, Beijing, China) and subsequently sequenced on an ABI-3730-XL DNA analyzer (Applied Biosystems, Foster City, CA, USA) utilizing the aforementioned amplification primers.

All newly generated sequences were deposited in GenBank (NCBI); corresponding accession numbers and detailed specimen information are summarized in Table S1. For phylogenetic analyses, available sequences of *Callistosporiaceae* and related families (*Asproinocybaceae*) were retrieved from GenBank. Besides outgroups, all downloaded sequences were screened to exclude those of insufficient length (e.g., ITS < 400 bp, LSU < 500 bp), with a high proportion of ambiguous bases, or from specimens with questionable identification (e.g., sequences labeled as “uncultured fungus”, lacking voucher information, or showing conflicting taxonomic assignments in preliminary BLAST searches). Outgroups were chosen following recent phylogenies of the *Agaricales*, with priority given to taxa with complete multi-locus data. After this quality filtering, taxa were selected based on (i) representation of major lineages within *Callistosporiaceae* and *Asproinocybaceae*, and (ii) preference for taxa with more available loci (ITS, LSU, *rpb2*, *tef1-α*) to minimize missing data. Due to extensive missing data in *rpb2* and *tef1-α* for many retained taxa, an additional phylogenetic tree based solely on ITS and LSU was constructed to confirm the placement of the new samples with a broader taxon sampling.

### 2.3. Phylogenetic Analyses

Raw trace files from bi-directional sequencing were assembled and manually edited using Sequencher v4.1.4 (Gene Codes Corp., Ann Arbor, MI, USA) to correct ambiguous bases. All subsequent dataset handling and phylogenetic analyses were integrated within the PhyloSuite v2 platform [21]. Newly generated sequences were combined with corresponding reference sequences downloaded from GenBank. Sequences for each locus were aligned using the “--auto” strategy in MAFFT v7.505 [22] and subsequently inspected and trimmed manually in BioEdit v7.7.1 [23].

To assess topological congruence, Maximum Likelihood (ML) trees were preliminarily inferred for each single-gene alignment. If no conflicting nodes were recovered with bootstrap support ≥75% in any of the single-gene trees (i.e., no mutually exclusive clades received ≥75% BS across different single-gene phylogenies), the four individual alignments were concatenated into a single combined dataset (ITS-LSU-*rpb2*-*tef1-α*), with missing sequences coded as “?”. Based on the established genetic relationships within the *Tricholomatineae*, *Lepista nebularis* (Batsch) Harmaja, *L*. *nuda* (Bull.) Cooke, and *Clitocybe dealbata* (Sowerby) P. Kumm. were selected as outgroups, all of which were represented by complete sequences for the four targeted loci. All phylogenetic analysis documents (including codes and settings) are provided as Supplementary File S1.

Best-fit models for each data partition were selected using the ModelFinder functionality implemented within IQ-TREE v3.0.1 [24,25] according to the Bayesian Information Criterion (BIC). The selected models were TVM + F + I + G4 for ITS, TN + F + I + R2 for LSU, TIM3 + I + G4 for *rpb2*, and TIM2e + I + G4 for *tef1-α*. The ML phylogeny of the concatenated dataset was inferred using IQ-TREE under an edge-linked proportional partition model with 1000 standard bootstrap replicates. Bayesian Inference (BI) was conducted in MrBayes v3.2.7a [26]. Since the best-fit models selected for IQ-TREE are not directly implementable in MrBayes, the general time-reversible model with invariant sites and gamma-distributed rate heterogeneity (GTR + I + G) was applied to all partitions with unlinked substitution parameters, following common practice in phylogenetic studies. Two parallel runs with four Markov chains were executed for 2,000,000 generations, sampling every 1000th generation. Convergence was assessed by ensuring the average standard deviation of split frequencies < 0.01 and effective sample sizes (ESS) > 200 for all parameters after discarding the first 25% as burn-in. Lastly, pairwise intergeneric genetic distances were calculated using the uncorrected p-distance method in MEGA11 [27], with pairwise deletion of gaps and missing data. All other parameters were kept at their default settings.

## 3. Results

### 3.1. Phylogenetic Results

**Figure 1:**
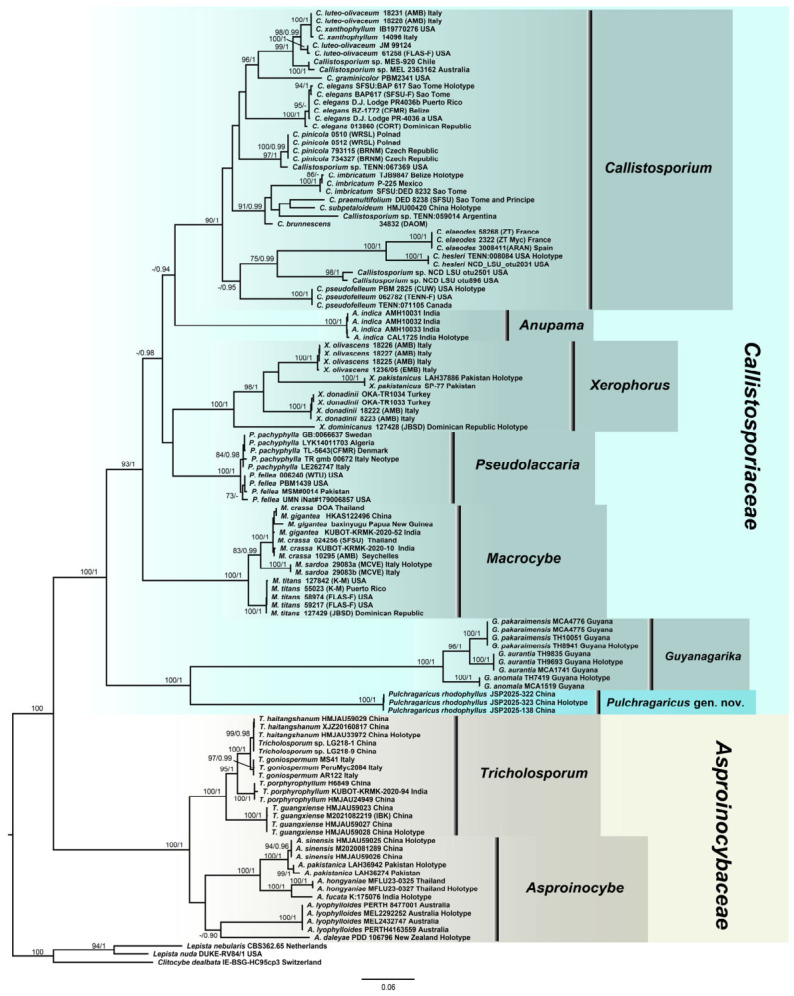
Phylogenetic relationships among representative genera in *Callistosporiaceae* and *Asproinocybaceae* inferred from a multigene dataset (ITS-LSU-*rpb2*-*tef1-α*) using both ML and BI. Only the ML tree is shown. Support values are indicated on branches as ML bootstrap (BS)/Bayesian posterior probability (PP); BS > 70% and PP > 0.90 are indicated on branches. Sequences derived from type specimens (holotype or neotype) are labeled accordingly. The phylogenetic tree was rooted with *Lepista nebularis*, *L. nuda*, and *Clitocybe dealbata*. A new genus and species are highlighted in cyan. The proportion of missing data for each gene partition is summarized in Table S1.

No topological inconsistencies were detected either among the single-gene trees (ITS, LSU, *rpb2*, and *tef1-α*) and the concatenated multigene datasets (ITS-LSU and ITS-LSU-*rpb2*-*tef1-α*), or between the ML and BI analyses for concatenated multigene datasets (see detailed in Figure S1A–D). Consequently, only the phylogenetic tree inferred from the ML analysis is presented, with statistical support values from both ML (bootstrap support, BS) and BI (posterior probabilities, PP) displayed on the respective nodes (Figure 1). Given the limited availability of *rpb2* and *tef1-α* sequences for certain reference taxa, a combined tree based solely on the ITS and LSU dataset is also provided in Figure S1E.

For the four-gene concatenated matrix, a total of 118 terminal taxa were assembled, comprising 106 sequences for ITS, 92 for LSU, 37 for *rpb2*, and 25 for *tef1-α*. The final aligned dataset comprised 4238 sites (including gaps), distributed as follows: 1018 bp for ITS, 1484 bp for LSU, 1056 bp for *rpb2*, and 680 bp for *tef1-α*.

Phylogenetic analyses (Figure 1) placed the newly discovered monophyletic lineage within the *Callistosporiaceae* with maximum statistical support (BS = 100%, PP = 1.00), positioning it as sister to the genus *Guyanagarika*. These results robustly confirm that our samples represent a novel genus and species within the family in the phylogenetic tree.

For the four-gene dataset, intergeneric distances were calculated using the uncorrected p-distance (Table 2). The divergence between *Pulchragaricus* and its phylogenetically closest relative, *Guyanagarika*, is 0.186 (18.6% nucleotide difference). This value falls within the range of intergeneric distances among well-established genera in *Callistosporiaceae* (min: *Anupama* vs. *Callistosporium* = 0.111; max: *Guyanagarika* vs. *Macrocybe* = 0.206). Furthermore, even the smallest distance between *Pulchragaricus* and any other genus in the dataset (*Pulchragaricus* vs. *Anupama* = 0.156) exceeds several accepted intergeneric distances, such as those between *Callistosporium* and *Macrocybe* (0.145) or *Callistosporium* and *Pseudolaccaria* (0.122). This indicates that the molecular divergence of *Pulchragaricus* is consistent with, or in some comparisons greater than, typical generic separation within these families. It should be noted that these p-distances were calculated from a concatenated alignment with a substantial proportion of missing data (e.g., *rpb2* and *tef1-α*), and are therefore considered as additional evidence. Although *Pulchragaricus* is currently monotypic and intrageneric distances could not be calculated, the substantial genetic gap separating it from its sister lineage, together with its phylogenetic distinctness and the morphological characteristics discussed below, supports its recognition at the generic rank.

### 3.2. Taxonomy

***Pulchragaricus*** S.P. Jian **gen. nov.**

MycoBank MB863543

Type species: *Pulchragaricus rhodophyllus* S.P. Jian sp. nov.

Etymology: *Pulchragaricus* is derived from the Latin adjective stem *pulchr-* (from *pulcher*, beautiful) and the generic name *Agaricus*, referring to the beautiful basidiomata that resemble species of *Agaricus*.

Diagnosis: *Pulchragaricus* is distinguished from the phylogenetically closest genera *Guyanagarika* and *Macrocybe* by the following combination of characteristics: tricholomatoid and medium-sized basidiomata, a tomentose and yellowish brown pileus with an involute margin, crowded, pink to pinkish lamellae, subclavate cheilocystidia, and broadly ellipsoid to ellipsoid basidiospores.

Description: Basidiomata tricholomatoid. Pileus—hemispherical to convex; surface—tomentose, yellowish brown; margin—involute; context—white. Lamellae—crowded, adnate, pink to pinkish. Stipe—central and solid; basal mycelium—white. Basidiospores—broadly ellipsoid to ellipsoid, smooth, inamyloid. Cheilocystidia—rare and subclavate. Pleurocystidia—absent. Clamp connections present in all parts.

**Figure 2:**
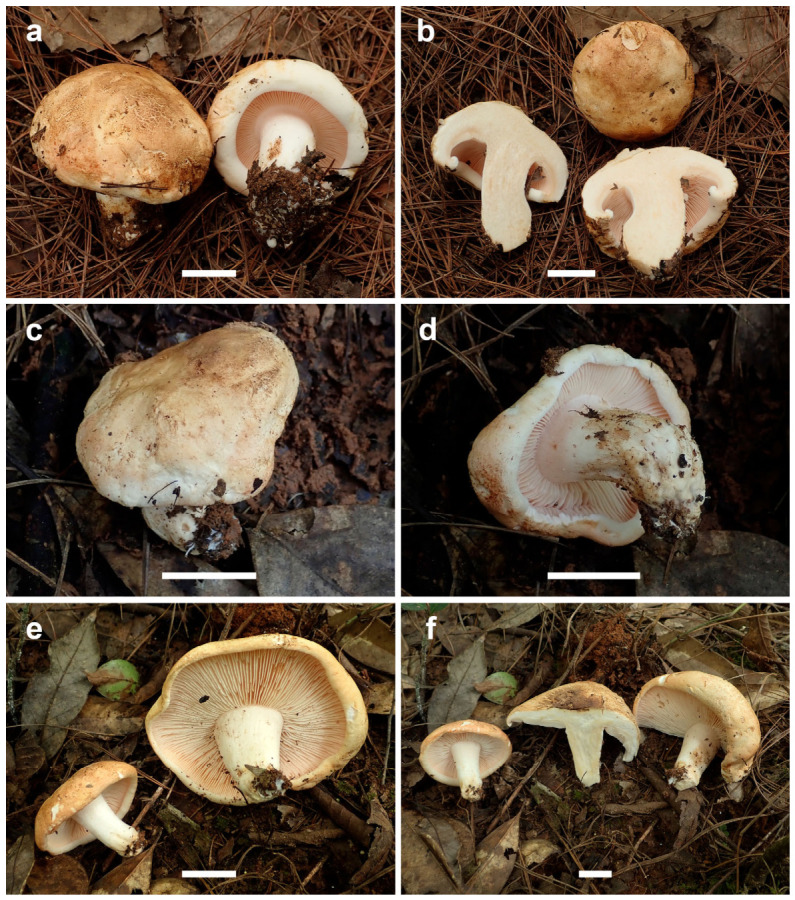
Fresh basidiomata of *Pulchragaricus rhodophyllus*. Colors of basidiomata are as recorded in the field (Pu’er City, China) under natural light conditions. KUN-HKAS 154740: (**a**) pileus, lamellae, and stipe; (**b**) context. KUN-HKAS 154741: (**c**) pileus; (**d**) lamellae and stipe. KUN-HKAS 154742 (holotype): (**e**) pileus, lamellae, and stipe. (**f**) Context. Scale bars = 2 cm.

**Figure 3:**
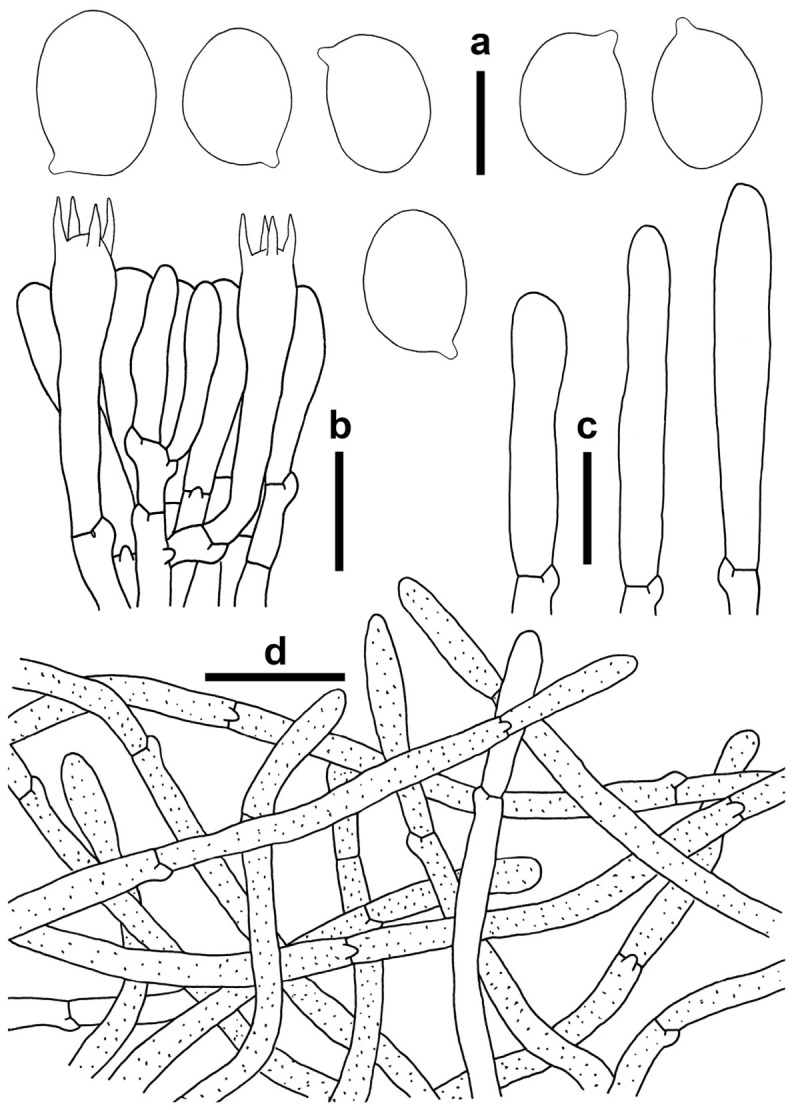
Microscopic structures of *Pulchragaricus rhodophyllus* (KUN-HKAS 154742, holotype). (**a**) Basidiospores. (**b**) Hymenium and subhymenium. (**c**) Cheilocystidia. (**d**) Pileipellis (suprapellis). All sections mounted in 5% KOH. Scale bars: 5 μm (**a**); 10 μm (**b**,**c**); 20 μm (**d**).

***Pulchragaricus rhodophyllus*** S.P. Jian **sp. nov.** Figure 2 and Figure 3.

MycoBank MB863544

Holotype: China, Yunnan Province, Pu’er City, Simao District, 17 km along Laosilan Road away from the city, 100°49′12.85″ E, 22°45′27.63″ N (datum WGS84, coordinate uncertainty: ca. 50 m), alt. 1254.3 m, scattered on soil, in the subtropical mixed forest (*Pinus kesiya* var. *langbianensis* and *Fagaceae*), 16 September 2025, collected by S.P. Jian & X.J. Xu, JSP2025-323 (KUN-HKAS 154742). GenBank: ITS = PZ267112; LSU = PZ229067; *rpb2* = PZ233666; *tef1-α* = PZ233669.

Etymology: “*rhodo*-” (rose) and “-*phyllus*” (leaf, here lamellae), referring to the pink-colored lamellae when young.

Diagnosis: *Pulchragaricus rhodophyllus* is distinguished from morphologically similar species of *Callistosporiaceae* by the following combination of characteristics: tricholomatoid basidiomata, yellowish brown pileus with involute margin, pink to pinkish lamellae, broadly ellipsoid basidiospores, sparse and subclavate cheilocystidia, and plentiful clamp connections.

Description: Basidiomata—medium-sized, tricholomatoid. Pileus—5.3–8.2 cm in diam., hemispherical to subhemispherical when young, then convex; surface—soft, tomentose, sometimes with finely net-like cracks at the center, brownish (#b7814b) to brown (#754e34) near the center, yellowish brown (#ecd19c) to faint yellow (#e5c7a1) towards the margin, sometimes uniformly pale brown (#ceb997); margin—persistently involute; context—1.5–2.1 cm thick at the center, white with yellowish (#e0d1b2) to dirty white (#dfdec1), unchanging in color when injured. Lamellae—crowded, emarginate to adnate, pink (#c78b65) when young, then pinkish (#c4af8b) to pale pink (#d5ae90), darkening after drying, edge entire and concolorous or somewhat paler, lamellulae numerous, unchanging in color when injured. Stipe—3.7–5.0 × 1.2–1.9 cm, central, tapering downwards, and solid; surface—glabrous, white (#f5f8ed) to whitish (#eff0df); context—concolorous with pileus context; basal mycelium—white (#adb6b2). Its odor is fragrant, and its taste is mild.

Basidiospores: [65/3/2] 6.5–8 (8.5) × 4.5–6 (6.5) μm, L_m_ × W_m_ = 7.03 (±0.42) × 5.16 (±0.34) μm, Q = 1.20–1.60, Q_m_ = 1.37 ± 0.09, hyaline in KOH, broadly ellipsoid to ellipsoid in side and face view, smooth, thin-walled, inamyloid. Basidia: 35–41 × 7–10 μm, clavate, four-spored; sterigmata 4–8 μm long. Cheilocystidia: sparse, 45–60 × 7–9 μm, cylindrical to subclavate, thin-walled, and fragile. Pleurocystidia: absent. Hymenophoral trama: more or less bilateral (divergent), made of cylindrical, parallel, smooth hyphae 4–8 μm wide, with yellow to yellowish-brown pigments; thromboplera (oleiferous hyphae sensu Clémençon [28]): abundant, luminous yellow. Pileipellis: usually two-layered. Suprapellis: about 90–160 μm thick, consisting of loose, erect, smooth, thin-walled, interwoven or irregularly arranged hyphae with yellowish-brown pigments, 4–9 μm wide. Subpellis—made up of subregular to regular, compactly arranged, thin-walled, hyaline, smooth, and cylindrical hyphae 5–12 μm wide, with abundant luminous yellow thromboplera; pileal trama—interwoven or irregular, composed of hyaline, thin-walled, cylindrical hyphae with a diameter of 5–10 (17) μm, occasionally distributed with white to yellowish, reflective thromboplera. Stipitipellis is a cutis composed of regular, compactly arranged, thin-walled, and hyaline hyphae with a diameter of (3.5) 4–13 (15) μm; stipe trama composed of regular, compactly arranged, thin-walled, and yellowish-brown hyphae 5–16 μm, with luminous yellow thromboplera. Caulocystidia—absent. Clamp connections present in all parts.

Ecology and distribution: Solitary or scattered on soil under the subtropical mixed forests (*Pinaceae* & *Fagaceae*). Known from Yunnan Province, China, fruiting from July to September.

Additional specimens examined: China, Yunnan Province, Pu’er City, Simao District, 17 km along Laosilan Road away from the city, 100°49′15.18″ E, 22°45′27.85″ N (datum WGS84, coordinate uncertainty: ca. 50 m), alt. 1303.5 m, scattered on soil, in the subtropical pine forest (dominated by *Pinus kesiya* var. *langbianensis*), 15 July 2025, collected by S.P. Jian & X.J. Xu, JSP2025-138 (KUN-HKAS 154740); *ibid.*, 100°49′13.22″ E, 22°45′28.00″ N (datum WGS84, coordinate uncertainty: ca. 50 m), alt. 1254.6 m, scattered on soil, in the subtropical mixed forest (*Pinus kesiya* var. *langbianensis* and *Fagaceae*), 16 September 2025, collected by S.P. Jian & X.J. Xu, JSP2025-322 (KUN-HKAS 154741).

Notes: *Pulchragaricus rhodophyllus* phylogenetically belongs to *Callistosporiaceae*, but it could be easily distinguished from species of other genera in this family (see Discussion for details). In general, *P. rhodophyllus* is morphologically more similar to *Agaricus fissuratus* F.H. Møller, *Guyanagarika pakaraimensis* Sánchez-García et al., *Lepista panaeolus* (Fr.) P. Karst., *Leucopaxillus alboalutaceus* (F.H. Møller & Jul. Schäff.) F.H. Møller, and *Pseudoclitopilus rhodoleucus* (Sacc.) Vizzini & Contu (synonym: *Leucopaxillus rhodoleucus* (Sacc.) Kühner). *Agaricus fissuratus* could be confused with *Pulchragaricus rhodophyllus* because both have a yellow pileus and pink lamellae. However, *A. fissuratus* differs from *P. rhodophyllus* by its radially cracked pileus, free lamellae, membranous ring, larger basidiospores, and spherical to broadly clavate cheilocystidia (7–45 × 5–27 μm) [29,30]. *Guyanagarika pakaraimensis* is characterized by tricholomatoid basidiomata and a solid, downwards-tapering stipe, which could be confused with *P. rhodophyllus*. Nevertheless, the orange pileus, pale yellow lamellae, longer stipe (5.5–10 cm long), and absence of cheilocystidia distinguish *G. pakaraimensis* from *P. rhodophyllus* [3].

*Lepista panaeolus* is another species similar to *P. rhodophyllus* in basidiomata size, with crowded pinkish-gray lamellae, and a robust stipe. However, the plano-convex to applanate pileus with brown marginal droplets, brownish lamellae, and warty basidiospores distinguishes *L. panaeolus* from *P. rhodophyllus* [31,32]. *Leucopaxillus alboalutaceus* is notable for its bitter taste, almost white pileus, and amyloid basidiospores with warty ornamentation, thus differing from *P. rhodophyllus* [33,34]. Lastly, *Pseudoclitopilus rhodoleucus* is a rare species distributed across Eurasia. It could be confused with *P. rhodophyllus* due to its pink to pinkish lamellae, robust stipe, and clamp connections. However, *Pseudoclitopilus rhodoleucus* has a white pileus, amyloid warty basidiospores, and lacks cheilocystidia [34,35,36].

## 4. Discussion

In the present four-locus dataset, only 37 of the 118 included taxa had sequences for *rpb2*, and 25 had sequences for *tef1-α*, resulting in a substantial proportion of missing data. It has been suggested that extensive missing data can sometimes produce inflated support values or misleading relationships [37]. However, several lines of evidence indicate that the missing sequences do not compromise the support for the new genus. Firstly, the clade corresponding to the new genus received maximum support (BS = 100%, PP = 1.0) in the four-locus dataset (Figure 1) and was consistent (BS = 100%) across four single-gene analyses (Figure S1A–D). Secondly, the new genus remained monophyletic with equally strong support (BS = 100%, PP = 1.0) when performing phylogenetic analysis using only a two-locus dataset (ITS-LSU), excluding the two most incomplete genes (*rpb2* and *tef1-α*) (Figure S1E). Thirdly, the missing sequences are not concentrated in any particular genus or lineage, but are instead scattered across the phylogeny (Table S1). Such scattered distribution of missing data has a limited impact on phylogenetic accuracy when the overall number of characters is large [38]. Taken together, the molecular delineation of the new genus is robust and is further corroborated by the morphological analysis, indicating that the phylogenetic result for this group is not an artifact of sparse gene sampling.

Species of *Callistosporiaceae* exhibit diverse colors, ranging from white, yellow, and brown to purple [1]. The trophic habits of species in this family are predominantly saprotrophic (on soil or rotten wood), with ectomycorrhizal symbiosis confirmed only in *Guyanagarika*. Numerous species of this family have been reported from Europe and the Americas, whereas species from China are rarely documented. In this study, we describe a new genus and species, *P*. *rhodophyllus*, which is a putatively ectomycorrhizal fungus based only on its occurrence in forests dominated by *Pinaceae* and *Fagaceae*. Direct anatomical evidence, such as Hartig net and mantle structures on host root tips, has yet to be obtained. Future studies employing root-tip sectioning or stable isotope tracing (e.g., ^13^C or ^15^N labeling) would help confirm the ectomycorrhizal status of this species and provide insights into the evolution of symbioses within *Callistosporiaceae*.

In the phylogenetic tree, *Pulchragaricus* is closely related to *Asproinocybe*, *Callistosporium*, *Guyanagarika*, and *Macrocybe*. However, it can be distinguished from all of them by a unique combination of characteristics: tricholomatoid, medium-sized basidiomata with a soft, yellowish-brown pileus and pinkish lamellae, subclavate cheilocystidia, and ellipsoid basidiospores. No single allied genus shares this entire suite of traits, which represents a clear morphological discontinuity supporting its recognition at the generic rank. Firstly, *Asproinocybe* was characterized by its pink to violet lamellae that turn reddish when injured, and tuberculate to stellate basidiospores, thus differing from *Pulchragaricus* [12,39,40]. *Callistosporium* has collybioid to pleurotoid habits and a smaller pileus (usually < 5 cm in diam.) that is yellow, brown, or brick red. It is lignicolous on rotting wood or wood debris, which significantly differs from *Pulchragaricus* [1,6,41]. Previously, *Guyanagarika* has been reported only from Guyana and is characterized by its tricholomatoid basidiomata, orange to dark orange pileus, and solid stipe. It differs from *Pulchragaricus* in having a pileus with an incurved to crenulate margin, pale yellow to orange lamellae, and lacking cheilocystidia [3]. *Macrocybe* is usually pantropically distributed and has a tricholomatoid habit. However, its larger basidiomata (>10 cm when mature), white, cream, or grayish pileus, and pale lamellae differ from those of *Pulchragaricus* [1,7].

Morphologically, *Pulchragaricus* could be confused with *Clitocybe* (Fr.) Staude, *Leucopaxillus*, *Pseudoclitopilus* Vizzini & Contu, and *Tricholoma* (Fr.) Staude, but is readily distinguished by the combination of a yellowish brown pileus, pink to pinkish lamellae that are emarginate to adnate, and inamyloid, smooth basidiospores. Specifically, *Clitocybe* differs in its white to yellowish pileus, decurrent, white to yellowish lamellae [42]. *Leucopaxillus* has a white pileus, white, decurrent lamellae, and amyloid, warty basidiospores [34,43], while *Pseudoclitopilus*, though also possessing pinkish lamellae, shares with *Leucopaxillus* the decurrent lamellae and amyloid, warty basidiospores, thus clearly distinct from *Pulchragaricus* [35,36,44]. The last genus, *Tricholoma,* is distinguished by its colorful pileus (e.g., white, grayish, brown, or yellow), adnexed to emarginate lamellae that are white, yellow, or gray [43,45,46], differing from *Pulchragaricus*. A full comparison of these genera with their diagnostic characteristics is provided in Table 3.

One limitation of the present study should be noted: specimen KUN-HKAS 154740 was immature and was therefore excluded from basidiospore size statistics and other microscopic character measurements; consequently, the description of mature basidiospores is based on the remaining collections. Additional fully mature collections would help confirm the range of intraspecific variability (e.g., the coloration of the basidiomata and variability of cheilocystidia) reported here.

## 5. Conclusions

In summary, *Pulchragaricus* can be distinguished from closely related genera by both molecular phylogenetic evidence and a combination of morphological traits. The key characteristics of this new genus are as follows: tricholomatoid basidiomata, a tomentose and yellowish-brown pileus with an involute margin, crowded and pinkish lamellae, subclavate cheilocystidia, broadly ellipsoid to ellipsoid, inamyloid basidiospores, and clamp connections present throughout. It should be noted, however, that the current dataset is built upon a limited number of collections, that several included taxa are represented by incomplete loci, and that the ecology of *Pulchragaricus* remains unconfirmed. Although only a single species is described to date, continued fieldwork and more comprehensive integrative analyses are likely to reveal additional species diversity. An expanded taxon and characteristic sampling will also be essential to more rigorously assess the phylogenetic significance of *Pulchragaricus* within *Callistosporiaceae*.

## Figures and Tables

**Table 1:** Diversity, ecology, and distribution of currently recognized genera within the family *Callistosporiaceae*.

Genera	Habit *	Substrate	Location	References
*Anupama*	Tricholomatoid	Terricolous	India	Raj et al. [4]
*Callistosporium*	Collybioid-pleurotoid	Lignicolous	Eurasia, Americas, and North Africa	Singer [6]
*Guyanagarika*	Tricholomatoid	Ectomycorrhizal	Guyana	Sánchez-García et al. [3]
*Macrocybe*	Tricholomatoid	Terricolous	Subtropical to tropical areas	Pegler et al. [7]
*Pseudolaccaria*	*Laccaria*-like	Terricolous	Eurasia and USA	Lavorato et al. [9]
*Xerophorus*	Collybioid	Terricolous	Europe and Greater Antilles	Vizzini et al. [1]

Notes: * For detailed terminology regarding basidiomata habit, see Noordeloos [10].

**Table 2:** Intergeneric uncorrected p-distances among genera of *Callistosporiaceae* based on the four-gene dataset.

	*Anupama*	*Callistosporium*	*Guyanagarika*	*Macrocybe*	*Pseudolaccaria*	*Pulchragaricus*	*Xerophorus*
*Anupama*							
*Callistosporium*	0.111						
*Guyanagarika*	0.170	0.186					
*Macrocybe*	0.145	0.140	0.206				
*Pseudolaccaria*	0.112	0.122	0.200	0.140			
*Pulchragaricus*	0.156	0.173	0.186	0.204	0.196		
*Xerophorus*	0.115	0.129	0.187	0.151	0.121	0.184	

Notes: Uncorrected p-distances were calculated in MEGA11 using pairwise deletion of gaps. The dataset comprises the concatenated alignment of ITS, LSU, *rpb2*, and *tef1-α*; positions with less than 50% taxon coverage were excluded.

**Table 3:** Comparison of morphological characteristics among *Pulchragaricus* and morphologically similar or phylogenetically related genera.

Genera	Pileus	Lamellae	Spores	Cystidia
*Asproinocybe*	Convex, gray, brown, red to violet	Adnate, violaceous to violet, changing reddish when injured	Inamyloid, tuberculate to stellate	Absent or cheilocystidia/pleurocystidia
*Callistosporium*	Convex to applanate, yellow, brown to brick red	Emarginate, adnate to adnexed, cream, yellow to vinaceous	Inamyloid, smooth	Absent or cheilocystidia
*Clitocybe* s. str. *	Convex to applanate, white to yellowish brown	Decurrent, white, cream to yellowish	Inamyloid, smooth	Absent
*Guyanagarika*	Convex to plano-convex, orange to dark orange	Adnate to sub-sinuate, pale yellow to orange	Inamyloid, smooth	Absent
*Leucopaxillus*	Convex to slightly depressed, white to ochraceous	Adnate to decurrent, white to pale cream	Amyloid, warts	Absent or cheilocystidia/pleurocystidia
*Macrocybe*	Convex, umbonate to depressed, white, cream to grayish	Sinuate, pale	Inamyloid, smooth	Absent or Pseudocystidia
*Pseudoclitopilus*	Convex to slightly depressed, white	Decurrent, pinkish	Amyloid, warts	Absent
*Pulchragaricus*	Hemispherical to convex, yellowish brown	Emarginate to adnate, pink to pinkish	Inamyloid, smooth	Cheilocystidia
*Tricholoma*	Convex to applanate, white, gray, brown to yellow	Adnexed to emarginate, white, grayish to yellow	Inamyloid, smooth	Absent

Notes: * *Clitocybe* s. str. was recently restricted by He et al. [42].

## Data Availability

The sequence data generated in this study can be obtained from NCBI GenBank (http://www.ncbi.nlm.nih.gov/, accessed on 24 February 2026). The data included in this study can be made available by contacting the authors.

## Supplementary Materials

The following supporting information can be downloaded at https://www.mdpi.com/article/10.3390/life16060899/s1: Figure S1: Phylogenetic relationships among representative genera in *Callistosporiaceae* and *Asproinocybaceae* inferred from each individual gene (ITS, LSU, *rpb2*, and *tef1-α*, Figure S1A–D) using ML, and a multigene dataset (ITS-LSU, Figure S1E) using both ML and BI. Sequences derived from type specimens (holotype or neotype) are labeled accordingly; Figure S2: Sample location map (Yunnan, China). The sampling locations of basidiomata are indicated by the blue triangle; Table S1: Collection information of voucher specimens and GenBank accession numbers for sequences used in phylogenetic analyses. N or H in parentheses means the neotype or holotype specimen. Sequences newly generated in this study are shown in bold. Supplementary File S1: The original data of phylogenetic analysis in this research.
