# Supplementary material for: Pulchragaricus rhodophyllus gen. et sp. nov. (Callistosporiaceae, Agaricales) from Yunnan, China, Based on Morphological and Molecular Data
Source: Life (Basel). 2026 May 27;16(6):899. doi: 10.3390/life16060899 (PMC13301615; doi:10.3390/life16060899)
Supplement: Supplementary file 1 [file life-16-00899-s001.zip › Figure S1 A-E.pdf]

Figure S1 A. The phylogenetic tree of ITS using ML.

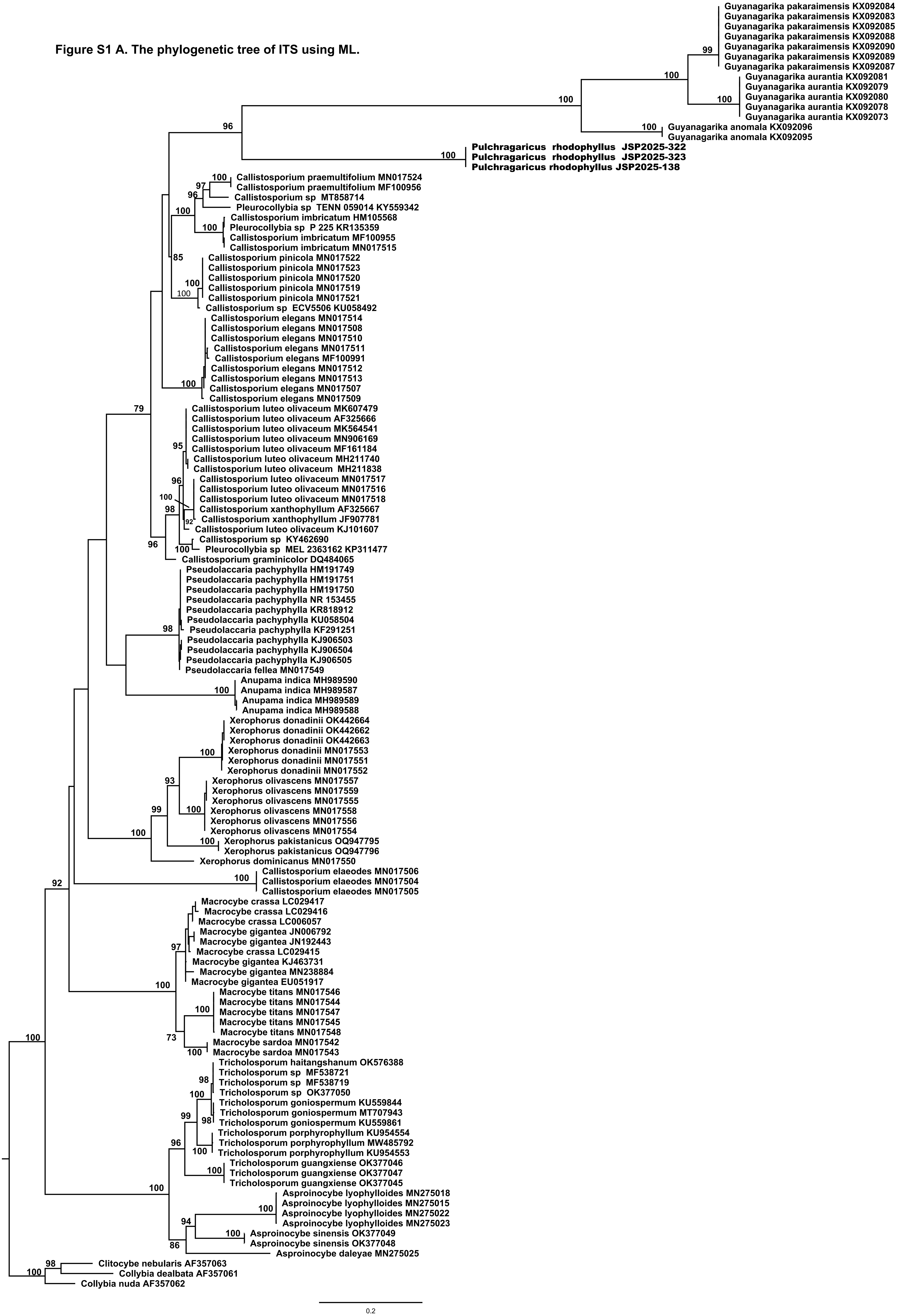

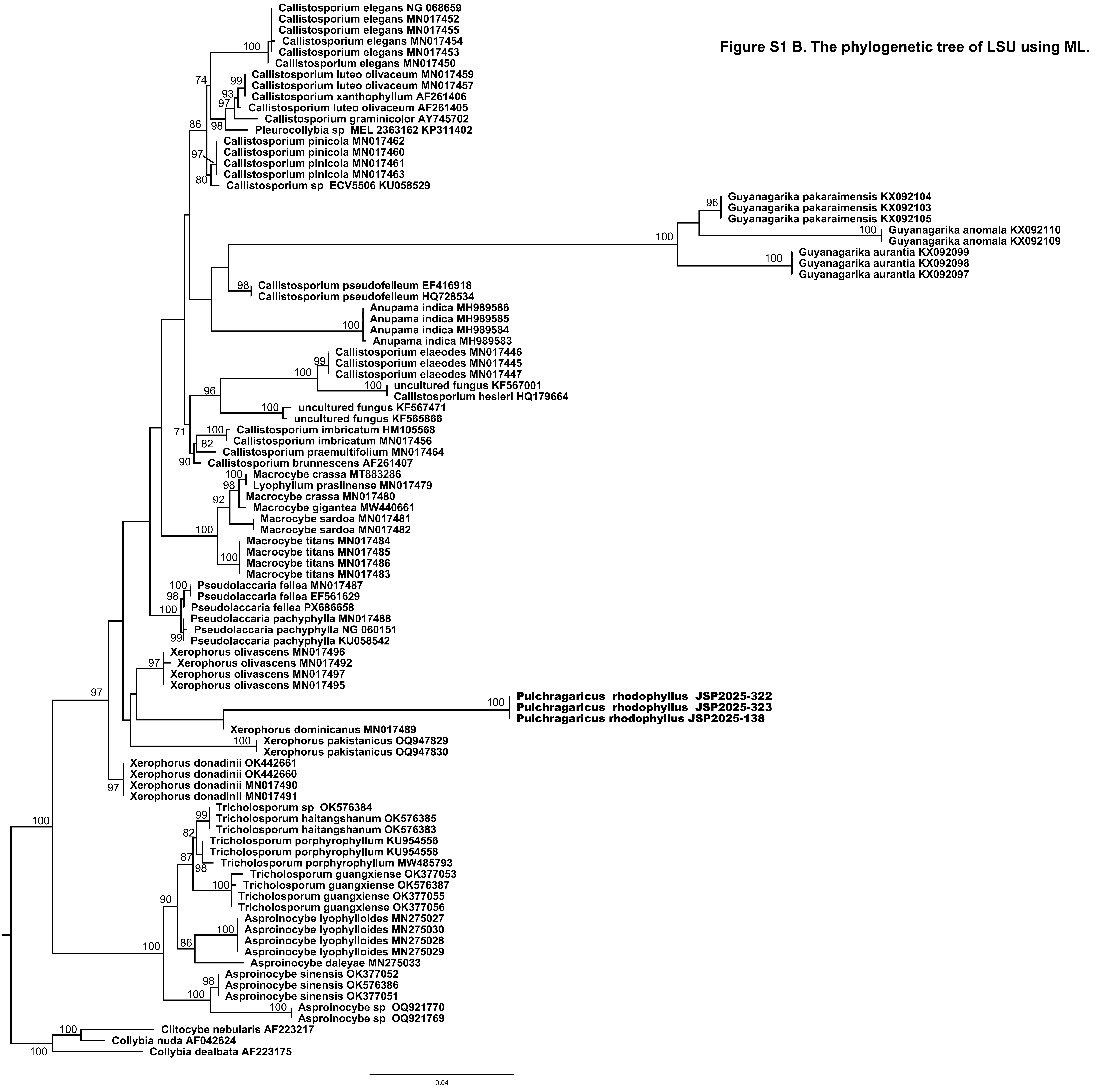

Figure S1 B. The phylogenetic tree of LSU using ML.

Figure S1 C. The phylogenetic tree of *rpb2* using ML.

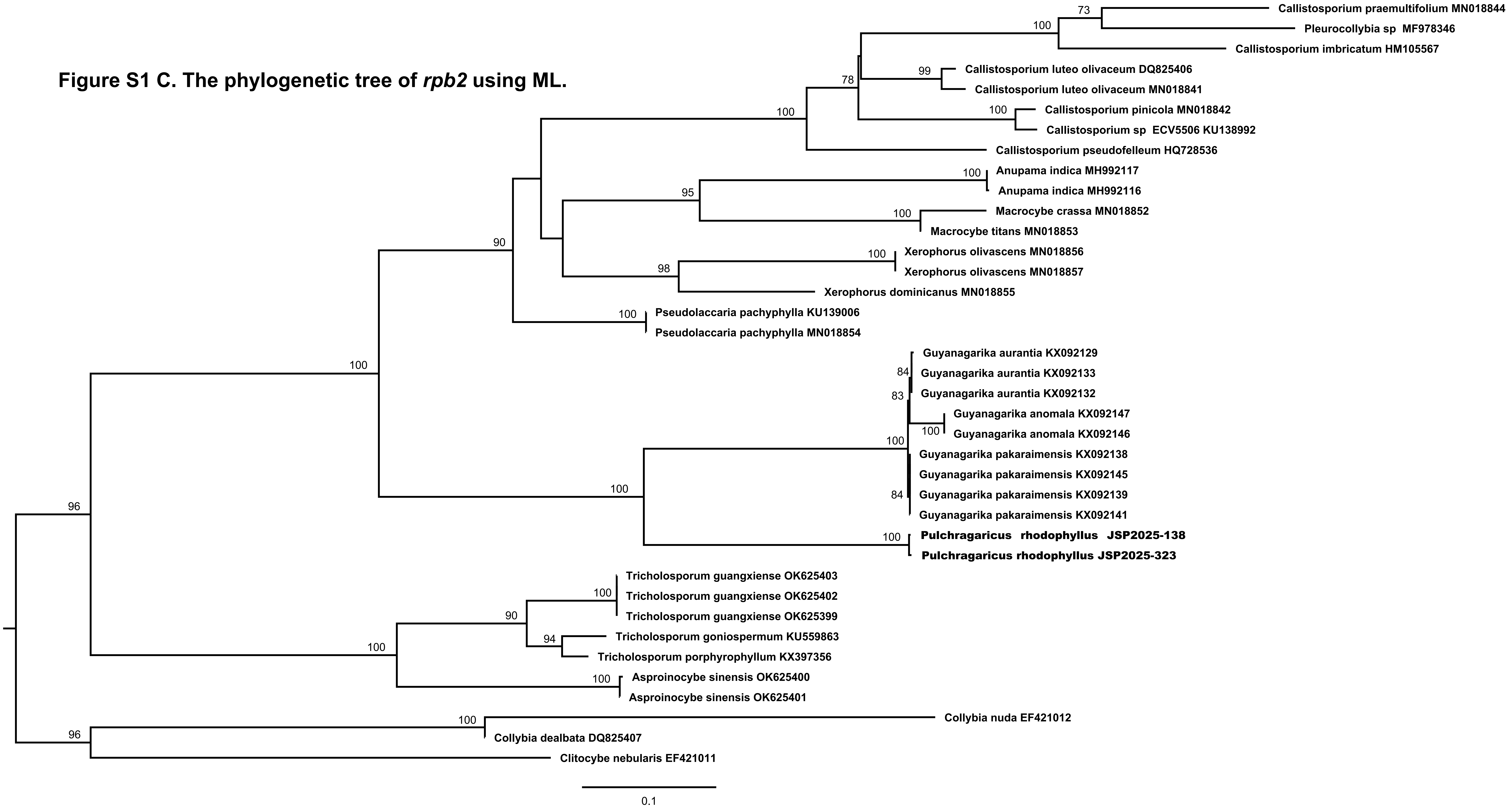

Figure S1 D. The phylogenetic tree of *tef1-α* using ML.

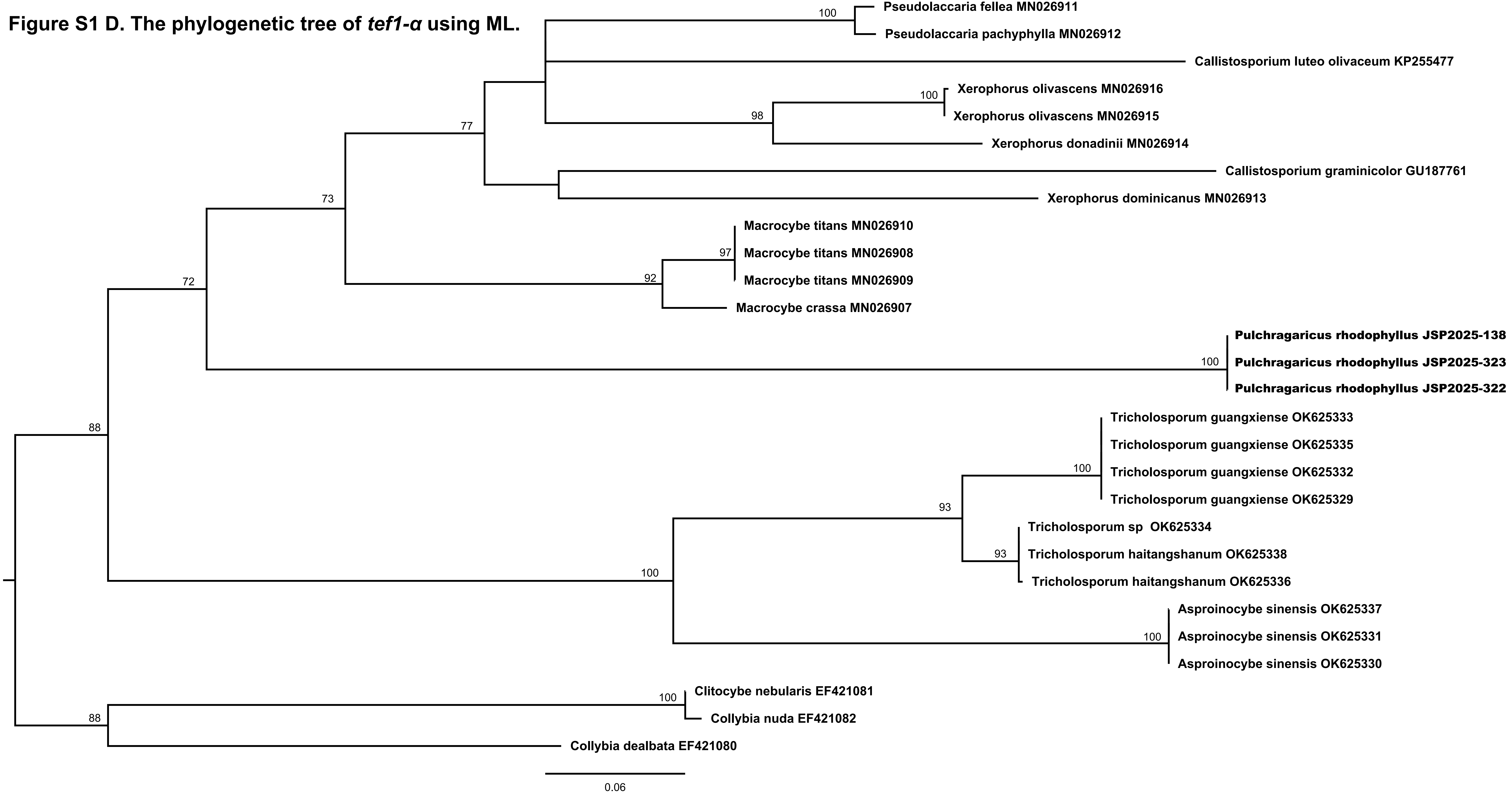

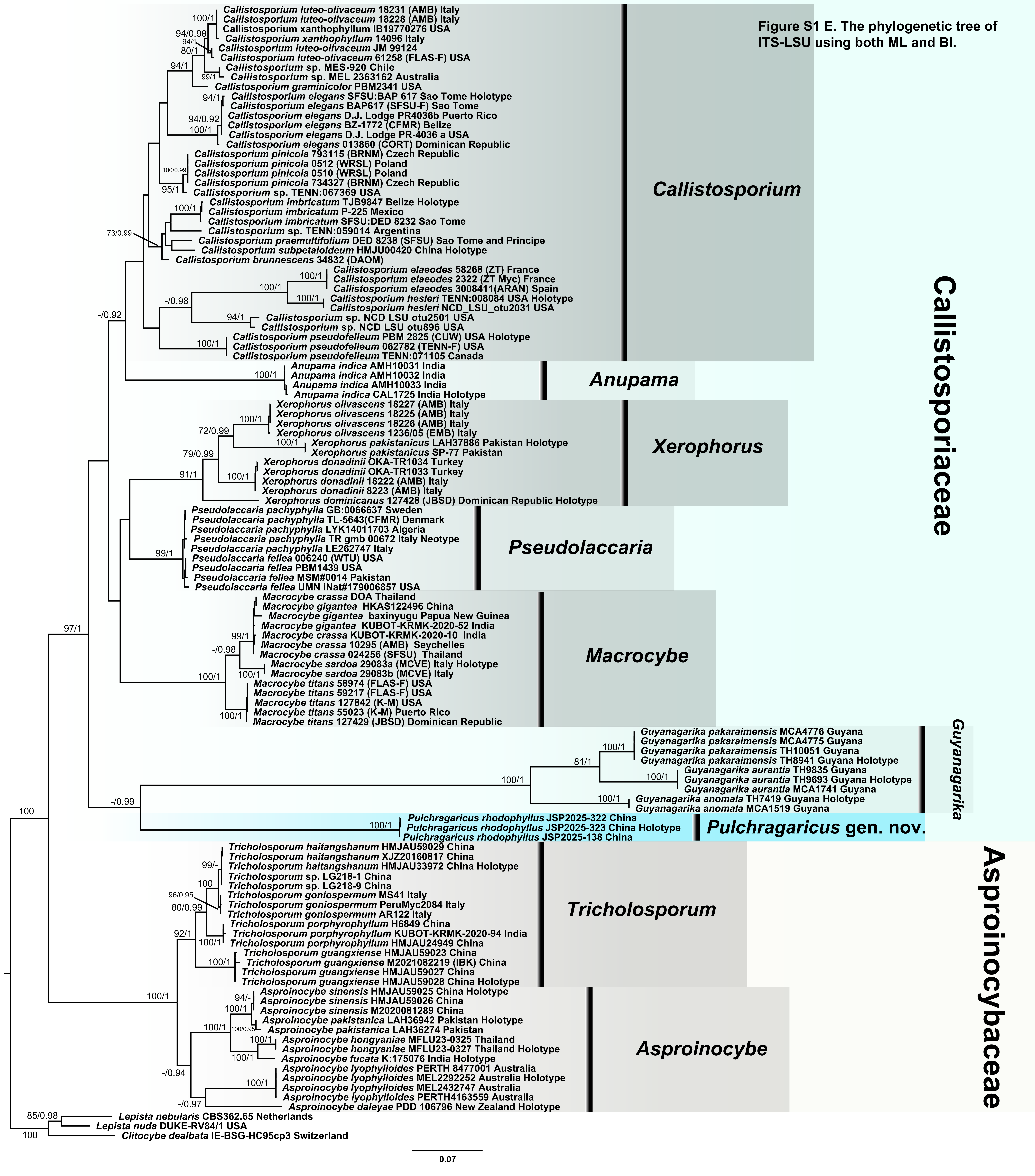

Figure S1 E. The phylogenetic tree of ITS-LSU using both ML and BI.
