# Supplementary material for: Pulchragaricus rhodophyllus gen. et sp. nov. (Callistosporiaceae, Agaricales) from Yunnan, China, Based on Morphological and Molecular Data
Source: Life (Basel). 2026 May 27;16(6):899. doi: 10.3390/life16060899 (PMC13301615; doi:10.3390/life16060899)
Supplement: Supplementary file 1 [file life-16-00899-s001.zip › Table S1.pdf]

life

MDPI

Table S1. Collection information of voucher specimens and GenBank accession numbers for sequences used in phylogenetic analyses. N or H in parentheses means the neotype or holotype specimen. Sequences newly generated in this study are shown in bold.

Species	Collection or collector no.	Locations	GenBank accession numbers			
			ITS	LSU	*rpb2*	*tef1-α*
Anupama indica	AMH10033	India	MH989590	MH989586	MH992117	-
Anupama indica (H)	CAL1725	India	MH989587	MH989583	-	-
Anupama indica	AMH10031	India	MH989588	MH989584	MH992116	-
Anupama indica	AMH10032	India	MH989589	MH989585	-	-
Asproinocybe daleyae (H)	PDD 106796	New Zealand	MN275025	MN275033	-	-
Asproinocybe fucata (H)	K:175076	India	MT921456	-	-	-
Asproinocybe lyophylloides	PERTH4163559	Australia	MN275018	MN275027	-	-
Asproinocybe lyophylloides	MEL2432747	Australia	MN275015	MN275028	-	-
Asproinocybe lyophylloides (H)	MEL2292252	Australia	MN275016	MN275029	-	-
Asproinocybe lyophylloides	PERTH 8477001	Australia	MN275022	MN275030	-	-
Asproinocybe sinensis (H)	HMJAU59025	China	OK377049	OK377052	OK625400	OK625330
Asproinocybe sinensis	HMJAU59026	China	OK377048	OK377051	OK625401	OK625331
Asproinocybe sinensis	M2020081289	China	-	OK576386	-	OK625337
Asproinocybe pakistanica (H)	LAH36942	Pakistan	MW969760	-	-	-
Asproinocybe pakistanica	LAH36274	Pakistan	MW969761	-	-	-
Asproinocybe hongyaniae	MFLU23-0325	Thailand	OR336172	OQ921770	-	-
Asproinocybe hongyaniae (H)	MFLU23-0327	Thailand	OR336171	OQ921769	-	-
Callistosporium brunnescens	34832 (DAOM)	-	-	AF261407	-	-
Callistosporium elaeodes	3008411(ARAN)	Spain	MN017506	MN017447	-	-
Callistosporium elaeodes	58268 (ZT)	France	MN017505	MN017446	-	-
Callistosporium elaeodes	2322 (ZT Myc)	France	MN017504	MN017445	-	-
Callistosporium elegans	D.J. Lodge PR-4036 a	USA	MN017513	MN017454	-	-

Table S1. *Cont.*

Species	Collection or collector no.	Locations	GenBank accession numbers			
			ITS	LSU	*rpb2*	*tef1-α*
Callistosporium elegans (H)	SFSU:BAP 617	Sao Tome	MF100991	NG068659	-	-
Callistosporium elegans	D.J. Lodge PR4036b	Puerto Rico	MN017514	MN017455	-	-
Callistosporium elegans	BAP617 (SFSU-F)	Sao Tome	MN017511	MN017452	-	-
Callistosporium elegans	BZ-1772 (CFMR)	Belize	MN017512	MN017453	-	-
Callistosporium elegans	013860 (CORT)	Dominican Republic	MN017509	MN017450	-	-
Callistosporium graminicolor	PBM2341	USA	DQ484065	AY745702	KJ424369	GU187761
Callistosporium hesleri	NCD_LSU_otu2031	USA	-	KF567001	-	-
Callistosporium hesleri (H)	TENN:008084	USA	HQ179664	HQ179664	-	-
Callistosporium imbricatum (H)	TJB9847	Belize	HM105568	HM105568	HM105567	-
Callistosporium imbricatum	SFSU:DED 8232	Sao Tome	MF100955	MN017456	-	-
Callistosporium imbricatum	P-225	Mexico	KR135359	-	-	-
Callistosporium luteo-olivaceum	18231 (AMB)	Italy	MN017518	MN017459	-	-
Callistosporium luteo-olivaceum	JM 99124	-	AF325666	AF261405	DQ825406	KP255477
Callistosporium luteo-olivaceum	18228 (AMB)	Italy	MN017516	MN017457	MN018841	-
Callistosporium luteo-olivaceum	61258 (FLAS-F)	USA	MH211838	-	-	-
Callistosporium pinicola	734327 (BRNM)	Czech Republic	MN017521	MN017462	-	-
Callistosporium pinicola	793115 (BRNM)	Czech Republic	MN017522	MN017463	-	-
Callistosporium pinicola	0510 (WRSL)	Poland	MN017519	MN017460	MN018842	-
Callistosporium pinicola	0512 (WRSL)	Poland	MN017520	MN017461	-	-
Callistosporium praemultifolium	DED 8238 (SFSU)	Sao Tome and Principe	MN017524	MN017464	MN018844	-
Callistosporium pseudofelleum (H)	PBM 2825 (CUW)	USA	EF416919	EF416918	-	-
Callistosporium pseudofelleum	062782 (TENN-F)	USA	HQ728533	HQ728534	HQ728536	-
Callistosporium pseudofelleum	TENN:071105	Canada	KX897421	-	-	-
Callistosporium subpetaloideum (H)	HMJU00420	China	MT858714	-	-	-
Callistosporium xanthophyllum	IB19770276	USA	AF325667	AF261406	-	-

Table S1. *Cont.*

Species	Collection or collector no.	Locations	GenBank accession numbers			
			ITS	LSU	*rpb2*	*tef1-α*
Callistosporium xanthophyllum	14096	Italy	JF907781	-	-	-
Callistosporium sp.	NCD LSU otu2501	USA	-	KF567471	-	-
Callistosporium sp.	NCD LSU otu896	USA	-	KF565866	-	-
Callistosporium sp.	MES-920	Chile	KY462690	-	-	-
Callistosporium sp.	TENN:067369	USA	KU058492	KU058529	KU138992	-
Callistosporium sp.	MEL 2363162	Australia	KP311477	KP311402	-	-
Callistosporium sp.	TENN:059014	Argentina	KY559342	-	MF978346	-
Guyanagarika anomala (H)	TH7419	Guyana	KX092096	KX092110	KX092147	-
Guyanagarika anomala	MCA1519	Guyana	KX092095	KX092109	KX092146	-
Guyanagarika aurantia	TH9835	Guyana	KX092079	KX092099	KX092133	-
Guyanagarika aurantia (H)	TH9693	Guyana	KX092078	KX092098	KX092132	-
Guyanagarika aurantia	MCA1741	Guyana	KX092073	KX092097	KX092129	-
Guyanagarika pakaraimensis	MCA4776	Guyana	KX092085	KX092104	KX092139	-
Guyanagarika pakaraimensis	TH10051	Guyana	KX092087	KX092105	KX092141	-
Guyanagarika pakaraimensis	MCA4775	Guyana	KX092084	KX092103	KX092138	-
Guyanagarika pakaraimensis (H)	TH8941	Guyana	KT339200	-	KX092145	-
Macrocybe crassa	KUBOT-KRMK-2020-10	India	MT883354	MT883286	-	-
Macrocybe crassa	DOA	Thailand	LC029415	-	-	-
Macrocybe crassa	024256 (SFSU)	Thailand	MN017540	MN017480	-	MN026907
Macrocybe crassa	10295 (AMB)	Seychelles	MN017539	MN017479	MN018852	-
Macrocybe gigantea	baxinyugu	Papua New Guinea	MN238884	-	-	-
Macrocybe gigantea	HKAS122496	China	ON794326	-	-	-
Macrocybe gigantea	KUBOT-KRMK-2020-52	India	MW445915	MW440661	-	-
Macrocybe sardoa (H)	29083a (MCVE)	Italy	MN017542	MN017481	-	-
Macrocybe sardoa (H)	29083b (MCVE)	Italy	MN017543	MN017482	-	-

Table S1. *Cont.*

Species	Collection or collector no.	Locations	GenBank accession numbers			
			ITS	LSU	*rpb2*	*tef1-α*
Macrocybe titans	127429 (JBSD)	Dominican Republic	MN017547	MN017486	-	MN026910
Macrocybe titans	58974 (FLAS-F)	USA	MN017545	MN017484	-	MN026908
Macrocybe titans	59217 (FLAS-F)	USA	MN017546	MN017485	MN018853	MN026909
Macrocybe titans	127842 (K-M)	USA	MN017548	-	-	-
Macrocybe titans	55023 (K-M)	Puerto Rico	MN017544	MN017483	-	-
Pseudolaccaria fellea	006240 (WTU)	USA	MN017549	MN017487	-	MN026911
Pseudolaccaria fellea	PBM1439	USA	-	EF561629	-	-
Pseudolaccaria fellea	MSM#0014	Pakistan	KJ906504	-	-	-
Pseudolaccaria fellea	UMN iNat#179006857	USA	PX684753	PX686658	-	-
Pseudolaccaria pachyphylla (N)	TR gmb 00672	Italy	NR_153455	NG060151	-	-
Pseudolaccaria pachyphylla	GB:0066637	Sweden	KU058504	KU058542	KU139006	-
Pseudolaccaria pachyphylla	LYK14011703	Algeria	-	MN017488	MN018854	MN026912
Pseudolaccaria pachyphylla	LE262747	Italy	HM191749	-	-	-
Pseudolaccaria pachyphylla	TL-5643(CFMR)	Denmark	KF291251	-	-	-
Pulchragaricus rhodophyllus	**HKAS154740**	**China**	**PZ267110**	**PZ229065**	**PZ233665**	**PZ233667**
Pulchragaricus rhodophyllus	**HKAS154741**	**China**	**PZ267111**	**PZ229066**	-	**PZ233668**
***Pulchragaricus rhodophyllus* (H)**	**HKAS154742**	**China**	**PZ267112**	**PZ229067**	**PZ233666**	**PZ233669**
Tricholosporum goniospermum	AR122	Italy	KU559861	-	KU559863	-
Tricholosporum goniospermum	MS41	Italy	KU559844	-	-	-
Tricholosporum goniospermum	PeruMyc2084	Italy	MT707943	-	-	-
Tricholosporum guangxiense	HMJAU59023	China	OK377045	OK377053	OK625399	OK625329
Tricholosporum guangxiense	HMJAU59027	China	OK377046	OK377055	OK625402	OK625332
Tricholosporum guangxiense (H)	HMJAU59028	China	OK377047	OK377056	OK625403	OK625333
Tricholosporum guangxiense	M2021082219 (IBK)	China	-	OK576387	-	OK625335
Tricholosporum haitangshanum	HMJAU59029	China	OK377050	OK576384	-	OK625334

Table S1. *Cont.*

Species	Collection or collector no.	Locations	GenBank accession numbers			
			ITS	LSU	*rpb2*	*tef1-α*
Tricholosporum haitangshanum (H)	HMJAU33972	China	OK576388	OK576383	-	OK625338
Tricholosporum haitangshanum	XJZ20160817	China	-	OK576385	-	OK625336
Tricholosporum porphyrophyllum	HMJAU24949	China	KU954553	KU954556	KX397356	-
Tricholosporum porphyrophyllum	H6849	China	KU954554	KU954558	-	-
Tricholosporum porphyrophyllum	KUBOT-KRMK-2020-94	India	MW485792	MW485793	-	-
Tricholosporum sp.	LG218-1	China	MF538719	-	-	-
Tricholosporum sp.	LG218-9	China	MF538721	-	-	-
Xerophorus dominicanus (H)	127428 (JBSD)	Dominican Republic	MN017550	MN017489	MN018855	MN026913
Xerophorus donadinii	OKA-TR1034	Turkey	OK442664	OK442661	-	-
Xerophorus donadinii	OKA-TR1033	Turkey	OK442663	OK442660	-	-
Xerophorus donadinii	18222 (AMB)	Italy	MN017551	MN017490	-	MN026914
Xerophorus donadinii	8223 (AMB)	Italy	MN017552	MN017491	-	-
Xerophorus olivascens	18226 (AMB)	Italy	MN017558	MN017496	MN018856	MN026916
Xerophorus olivascens	18227 (AMB)	Italy	MN017559	MN017497	MN018857	-
Xerophorus olivascens	18225 (AMB)	Italy	MN017557	MN017495	-	MN026915
Xerophorus olivascens	1236/05 (EMB)	Italy	MN017554	MN017492	-	-
Xerophorus pakistanicus (H)	LAH37886	Pakistan	OQ947795	OQ947829	-	-
Xerophorus pakistanicus	SP-77	Pakistan	OQ947796	OQ947830	-	-
Outgroups						
Lepista nebularis	CBS362.65	Netherlands	AF357063	AF223217	EF421011	EF421081
Lepista nuda	DUKE-RV84/1	USA	AF357062	AF042624	EF421012	EF421082
Clitocybe dealbata	IE-BSG-HC95cp3	Switzerland	AF357061	AF223175	DQ825407	EF421080
