# Supplementary figures and images for: Pulchragaricus rhodophyllus gen. et sp. nov. (Callistosporiaceae, Agaricales) from Yunnan, China, Based on Morphological and Molecular Data
Source: Life (Basel). 2026 May 27;16(6):899. doi: 10.3390/life16060899 (PMC13301615; doi:10.3390/life16060899)

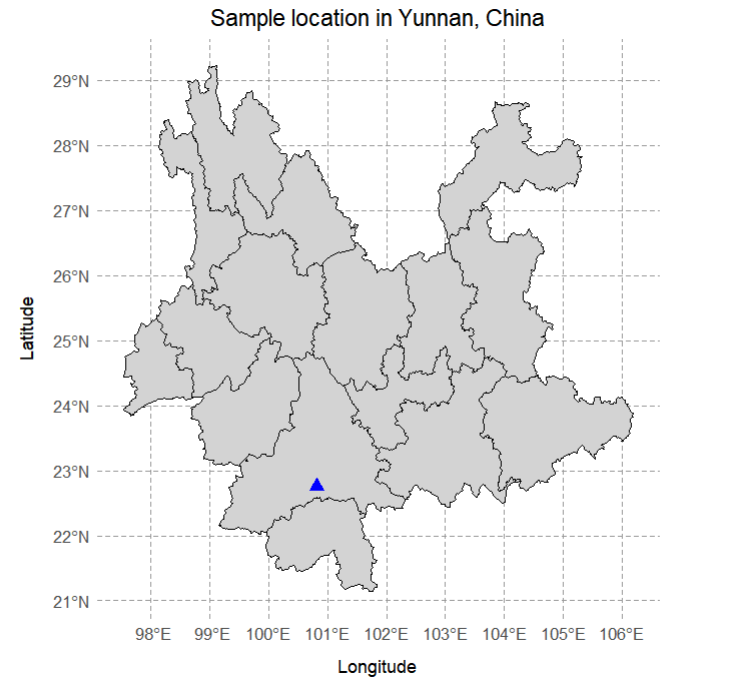

Supplement: Supplementary file 1 [file life-16-00899-s001.zip › Figure S2.png]
